# Differences in environmental preferences towards cycling for transport among adults: a latent class analysis

**DOI:** 10.1186/s12889-016-3471-5

**Published:** 2016-08-12

**Authors:** Lieze Mertens, Jelle Van Cauwenberg, Ariane Ghekiere, Ilse De Bourdeaudhuij, Benedicte Deforche, Nico Van de Weghe, Delfien Van Dyck

**Affiliations:** 1 Department of Movement and Sport Sciences, Faculty of Medicine and Health Sciences, Ghent University, Watersportlaan 2, B-9000 Ghent, Belgium; 2 Department of Public Health, Faculty of Medicine and Health Sciences, Ghent University, De Pintelaan 185, 4k3, B-9000 Ghent, Belgium; 3 Department of Human Biometry and Biomechanics, Faculty of Physical Education and Physical Therapy, Vrije Universiteit Brussel, Pleinlaan 2, B-1050 Brussels, Belgium; 4 Research Foundation Flanders (FWO), Egmontstraat 5, 1000 Brussels, Belgium; 5 Department of Geography, Faculty of Sciences, Ghent University, Krijgslaan 281, S8, B-9000 Ghent, Belgium

**Keywords:** Built environment, Biking, Adulthood, Subgroup, Photographs, Transport

## Abstract

**Background:**

Increasing cycling for transport can contribute to improve public health among adults. Micro-environmental factors (i.e. small-scaled street-setting features) may play an important role in affecting the street’s appeal to cycle for transport. Understanding about the interplay between individuals and their physical environment is important to establish tailored environmental interventions. Therefore, the current study aimed to examine whether specific subgroups exist based on similarities in micro-environmental preferences to cycle for transport.

**Methods:**

Responses of 1950 middle-aged adults (45–65 years) on a series of choice tasks depicting potential cycling routes with manipulated photographs yielded three subgroups with different micro-environmental preferences using latent class analysis.

**Results:**

Although latent class analysis revealed three different subgroups in the middle-aged adult population based on their environmental preferences, results indicated that cycle path type (i.e. a good separated cycle path) is the most important environmental factor for all participants and certainly for individuals who did not cycle for transport. Furthermore, only negligible differences were found between the importances of the other micro-environmental factors (i.e. traffic density, evenness of the cycle path, maintenance, vegetation and speed limits) regarding the two at risk subgroups and that providing a speed bump obviously has the least impact on the street’s appeal to cycle for transport.

**Conclusions:**

Results from the current study indicate that only negligible differences were found between the three subgroups. Therefore, it might be suggested that tailored environmental interventions are not required in this research context.

**Electronic supplementary material:**

The online version of this article (doi:10.1186/s12889-016-3471-5) contains supplementary material, which is available to authorized users.

## Background

Cross-sectional evidence has shown that active transport, especially cycling for transport, could be an important contributor to general public health by increasing physical activity (PA) levels among adults, reducing the risk of all-cause mortality and helping to maintain a healthy body weight [1, 2]. Cycling for transport can be integrated into adults’ daily life routines, is feasible and inexpensive, and can reduce traffic congestion and CO_2_ emissions [3–11]. As 50 % of all trips in Europe are shorter than 3 km, a feasible distance to cycle, there is considerable potential for an increase in the prevalence of cycling for transport [12]. Current Belgian statistics showed that, for adults, only 25 and 14 % of all trips shorter than 3 and 5 km respectively are undertaken using active transport (i.e. walking or cycling) [13]. Consequently, there is a need for interventions to promote cycling for transport in adults. In this regard, it is important to verify the key determinants of cycling for transport in adults.

Ecological models emphasize the importance of the physical environment, together with social and individual characteristics, to explain PA [14]. Furthermore, it is known that transport-related PA is more consistently associated with the physical environment than recreational physical activity [15]. Previous studies indicated that micro-environmental factors (e.g. evenness of the cycle path, vegetation, speed limits) might be more amenable to change than macro-environmental factors (e.g. street connectivity, residential density) [16, 17]. Since micro-environmental factors are relatively small-scale street-setting features and can be influenced on a neighborhood level by local actors, they are more feasible to target in existing neighborhoods than macro-environmental factors which are large-scale urban planning features influenced on regional/national level [16, 17]. Unfortunately, knowledge about the influence of these micro-environmental factors on adults’ cycling for transport is scarce and often inconsistent [18–22]. This is mainly due to the applied cross-sectional observational study designs [21, 23]; stronger designs with improved causal inference are necessary [21, 24–27]. A possible solution would be to conduct on-site experiments, but since these are usually long-term expensive projects, and since it is ethically not defensible to change real environments without being sure that these changes are effective (risk of negative effects and difficulty undoing real-life changes), another approach is required. Therefore, we developed a methodology using manipulated photographs which can simulate these experiments and identify critical environmental correlates associated with a street’s appeal to cycle for transport. This methodology studies the effects of environmental changes (manipulations) under controlled conditions, i.e. controlling the variation within and between the manipulated micro-environmental factors. Comparison with on-site responses [28, 29] support the validity of responses to colored photographs.

A recent large-scale conjoint study with manipulated photographs was able to identify the relative importance of a range of relevant displayed micro-environmental factors in the decision process of choosing the most appealing of two possible cycling routes [30]. The main finding was that the provision of cycle paths separated from motorized traffic is the best strategy to increase the street’s appeal to cycle for transport among middle-aged adults. Furthermore, this study showed that in streets where it is impossible to provide a well-separated cycle path (e.g. due to financial or space constraints), targeting micro-environmental factors related to safety (i.e. speed limit, traffic density) may be more effective in promoting bicycle transport than micro-environmental factors related to comfort (i.e. evenness of the cycle path surface) or aesthetics (i.e. vegetation, maintenance). On the other hand, micro-environmental factors related to comfort or aesthetics were more important in streets where a well-separated cycle path was already provided. However, we do not know whether these environmental changes are beneficial for the entire target population (i.e. middle-aged adults between 45 and 65 years old). In order to optimize environments and thus environmental interventions with the aim to encourage cycling for transport, it is important to gain insight in the associations of the physical environment (positive or negative) with cycling for transport among different subgroups [31].

Existing literature has revealed some different transportation patterns, needs, and purposes between different subgroups. For example, previous studies showed that issues of safety and comfort regarding cycling for transport are more important for women compared to men [32, 33]. Since the amount of cycling is determined by the inter-relation between individuals and their physical environment [14, 34], it is important to understand these interactions. First, it must be ascertained whether micro-environmental preferences towards cycling for transport are specific to particular subgroups, especially those who could benefit most from these interventions (i.e. at risk subgroups like those with poor attitudes towards cycling, poorer cycling skills or those living in a neighborhood with unsafe traffic conditions) [32, 33, 35, 36]. To create a mass cycling culture, it may be essential to target infrastructure and policies likely to influence groups that are currently not cycling a lot (e.g. women or older people) [37]. As it appears that regular cyclists will cycle regardless of the circumstances (e.g. lack of good cycling infrastructures, long travel distance) because they like to cycle [38], tailoring environmental interventions for at-risk subgroups should be possible without disadvantaging regular cyclists. Also, identifying the demographics and other characteristics of at-risk subgroups would enable the development of environmental interventions in environments most relevant to these populations.

Therefore, the current study aimed to examine whether there are subgroups with different micro-environmental preferences for cycling for transport among middle-aged adults (45–65 years) using latent class analysis. Furthermore, specific characteristics of these subgroups were identified based on socio-demographics, transport behavior, psychosocial determinants of cycling for transport, neighborhood environmental perceptions, cycling skills, concerns and preferences of participants.

## Methods

### Protocol and measures

Flemish middle-aged adults were recruited by purposeful convenience using email, social media, family, friends, clubs, organizations and companies. Furthermore, snowball sampling was used to recruit additional participants. As a wider age range might cause interference and therefore less accurate results [5], only adults between 45 and 65 years old were invited to participate in our research. This subgroup was selected as from the age of 45 years there is an increased risk of cardiovascular disease that may be partially attributed to an age-related decline in regular physical activity [1–3]. Older adults (>65 years) were not included in this research because they can be considered as a separate group due to their retirement and limited mobility in comparison to younger adults [4]. Eighteen participants who fell outside the age range of 45–65 years old, were excluded from the analysis. In total, 1950 middle-aged adults completed the two-part web-based questionnaire, developed with Sawtooth Software (SSI Web version 8.3.8.). Data collection took place between November 2014 and January 2015. Additional study details have been described elsewhere [30].

### The web-based questionnaire

The web-based questionnaire consisted of two main parts. In the first part, questions gathered information about participant characteristics as described below.

Self-reported *socio-demographic variables* included age, gender, educational level (two categories: primary school, lower/higher secondary - tertiary), area of residence (two categories: village, town or rural area - city or city border), weight and height (to calculate body mass index).

Participants’ *transport behavior* (i.e. walking and cycling for transport), cycling for leisure and motorized transport were assessed using the relevant sections of the validated International Physical Activity Questionnaire (IPAQ long form: ‘usual week’) [39, 40].

*Psychosocial determinants* focusing on cycling for transport were assessed based on validated questionnaires of psychosocial correlates of general physical activity [41] and psychosocial correlates of cycling-specific behaviors among adults [42, 43]. Seven psychosocial correlates of cycling for transport (5-point scale) were generated (see Table 1).

**Table 1:** Differences in socio-demographics, transport behavior, perceptions, cycling skills, opinions and psychosocial determinants between the subgroups

	Total sample	Subgroup 1	Subgroup 2	Subgroup 3	*p-value*
*Segment Sizes (n)*	*n* = 1950	*n* = 232	*n* = 598	*n* = 1120	
	100 %	11.9 %	30.7 %	57.4 %	
*Socio-demographic characteristics*					
Age (yrs, M ± SD)	54.3 ± 5.6	54.7 ± 5.5	54.1 ± 5.5	54.3 ± 5.7	0.328
Gender (% women)	56.8	47.8	60.9	56.5	0.003
SES (% tertiary education)	64.6	68.1	65.2	63.5	0.376
Area of residence (% village, town or rural)	59.4	54.3	56.7	62.0	0.025
BMI (kg/m^2^)	25.2 ± 3.8	25.3 ± 4.2	25.2 ± 3.8	25.1 ± 3.7	0.732
Cohabitation (%)	86.1	81.9	85.2	87.4	0.064
*Transport behavior*					
Motorized transport min/wk (M ± SD)	215.1 ± 252.2	210.9 ± 268.9	201.9 ± 227.6	223.0 ± 260.8	0.258
Bicycle transport min/wk (M ± SD)	147.7 ± 171.1	178.8 ± 181.3^b,c^	135.1 ± 147.6^a^	148.0 ± 179.7^a^	0.005
Walk for transport min/wk (M ± SD)	63.5 ± 109.4	81.9 ± 136.3^b,c^	63.6 ± 110.0^a^	59.7 ± 102.4^a^	0.021
Bicycle leisure time min/wk (M ± SD)	120.3 ± 170.9	132.6 ± 174.2	114.5 ± 166.5	120.8 ± 172.5	0.397
Number of motorized vehicles (M ± SD)	1.6 ± 1.0	1.3 ± 0.9^b,c^	1.6 ± 1.0^a^	1.6 ± 1.0^a^	<0.001
*Psychosocial determinants (5-point scale) (M ± SD)*					
Habit (1 item)	3.4 ± 1.5	3.7 ± 1.5^b,c^	3.3 ± 1.5^a^	3.3 ± 1.5^a^	0.009
Social norm (4 items, α = 0.90)	2.9 ± 1.2	3.0 ± 1.3	2.9 ± 1.2	2.8 ± 1.1	0.178
Modeling (4 items, α = 0.55)	3.2 ± 0.8	3.3 ± 0.8^c^	3.2 ± 0.8	3.1 ± 0.8^a^	0.046
Social support (4 items, α = 0.81)	2.4 ± 1.0	2.6 ± 1.0	2.4 ± 1.0	2.4 ± 0.9	0.184
Self-efficacy (11 items, α = 0.92)	3.7 ± 0.9	3.9 ± 0.9^b,c^	3.7 ± 0.9^a^	3.7 ± 0.9^a^	0.006
Perceived benefits (10 items, α = 0.84)	4.0 ± 0.6	4.1 ± 0.6^b,c^	4.0 ± 0.6^a^	4.0 ± 0.6^a^	0.008
Perceived barriers (16 items, α = 0.90)	2.4 ± 0.7	2.3 ± 0.7^b,c^	2.4 ± 0.7^a^	2.4 ± 0.7^a^	0.014
*Perceived neighborhood environment (5-point scale) (M ± SD)*					
Amount of single unit houses	3.0 ± 1.5	2.7 ± 1.5^b,c^	3.0 ± 1.5^a^	3.0 ± 1.4^a^	0.003
Presence of shops in the neighborhood	3.3 ± 1.4	3.4 ± 1.3	3.2 ± 1.4	3.3 ± 1.3	0.168
Presence of a stop for public transport	4.3 ± 1.0	4.4 ± 1.0	4.3 ± 1.0	4.3 ± 1.0	0.464
Presence of recreational opportunities (park, pool)	3.3 ± 1.4	3.5 ± 1.4	3.3 ± 1.4	3.3 ± 1.4	0.134
Neighborhood traffic safety	3.2 ± 1.1	3.3 ± 1.1	3.1 ± 1.1	3.2 ± 1.1	0.168
Neighborhood safety of crime	2.2 ± 1.0	2.1 ± 1.0	2.1 ± 1.0	2.2 ± 1.0	0.391
Sufficient cycling infrastructure	3.1 ± 1.1	3.0 ± 1.1	3.1 ± 1.1	3.1 ± 1.1	0.551
Neighborhood social environment	3.4 ± 1.0	3.4 ± 1.0	3.4 ± 1.0	3.4 ± 1.0	0.559
Good maintenance of cycling infrastructure	2.9 ± 1.1	2.8 ± 1.1	2.9 ± 1.1	2.9 ± 1.1	0.415
Presence of vegetation	3.1 ± 1.1	3.1 ± 1.1	3.1 ± 1.1	3.1 ± 1.1	0.939
*Cycling skills (5-point scale) (M ± SD)*	4.1 ± 0.7	4.1 ± 0.8	4.1 ± 0.7	4.1 ± 0.7	0.963
*Cycling concerns (5-point scale) (M ± SD)*					
As a cyclist I feel vulnerable in the traffic	3.7 ± 0.9	3.9 ± 0.9^b^	3.7 ± 1.0^a,c^	3.7 ± 0.9^b^	0.007
Importance of a fluorescent vest or bicycle helmet	4.9 ± 1.8	4.9 ± 1.8	4.7 ± 1.8^c^	5.0 ± 1.8^b^	0.001
*Cycling preferences (5-point scale) (M ± SD)*					
I prefer the safest cycling route	3.8 ± 0.9	3.9 ± 1.0^b^	3.7 ± 0.9^a,c^	3.9 ± 0.9^b^	0.002
I prefer the shortest cycling route	3.2 ± 1.0	3.2 ± 1.0	3.2 ± 1.0	3.2 ± 0.9	0.670
I prefer the most beautiful cycling route	3.6 ± 0.9	3.6 ± 0.9	3.6 ± 0.9	3.6 ± 0.9	0.215
I prefer to cycle alone	3.6 ± 1.0	3.7 ± 1.0^c^	3.7 ± 1.0^c^	3.5 ± 1.1^a,b^	0.003

^a^signficiant difference with subgroup 1

^b^significant difference with subgroup 2

^c^significant difference with subgroup 3

*Perceptions of the physical neighborhood* environment were evaluated using the validated Assessing Levels of Physical Activity (ALPHA) environmental questionnaire [44, 45]. Ten items assessed participants’ perceptions of their neighborhood environment using a 5-point scale ranging from totally disagree to totally agree (see Table 1).

Lastly, participants described their perceived *cycling skills and concerns and preferences* about cycling for transport using a five-point scale (1 = totally disagree; 5 = totally agree) inspired by a previously used questionnaire assessing basic cycling skills among children [46]. The construct cycling skills was created using the following two items: ‘I think I can cycle well’ and ‘I find cycling on a straight line or with one hand easy’ (α = 0.77). Furthermore, four separate items assessed the preferences for cycling for transport and two assessed cycling concerns (see Table 1). For the construct ‘I find a fluorescent vest or bicycle helmet important’, a sum was made between: ‘I find wearing a fluorescent vest or bicycle helmet important’ and ‘I wear a fluorescent vest or bicycle helmet’ (α = 0.81).

**Fig. 1:**
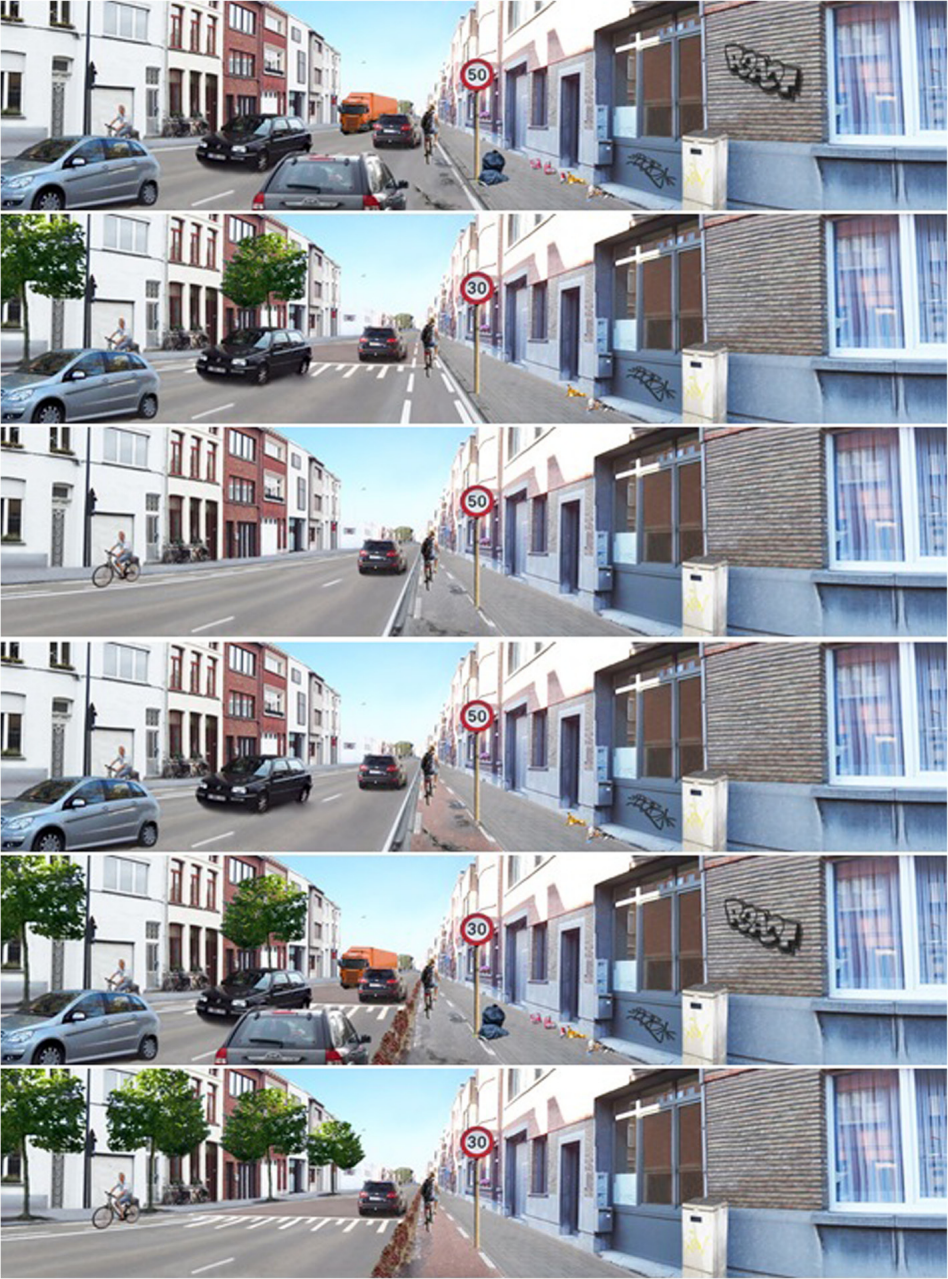
Examples of manipulated photographs, differing in seven micro-environmental factors with a maximum of 6 level. These examples ranging from the least separated cycle path (first photograph) to the most separated cycle path (last photograph) and randomly differed in the other micro-environmental factors

In the second part of the web-based questionnaire, a series of twelve randomly assigned choice tasks were presented to the participants using manipulated photographs to illustrate two possible routes to cycle along. For each choice task, participants had to choose which of the two depicted streets (manipulated photographs) they would prefer to cycle along to the house of their riend. This choice based conjoint (CBC) method [47] enabled examination of the characteristics influencing a street’s appeal to cycle for transport. Each manipulated photograph was different in one to seven micro-environmental factors, which varied in two to six levels (see Fig. 1): traffic density (3 levels), vegetation (3 levels), speed limit (2 levels), speed bump (2 levels), type of cycle path (6 levels), maintenance (3 levels) and evenness of the cycle path (3 levels). The selection of these micro-environmental factors was based on existing literature [15, 48] and previous research with (non-) manipulated panoramic photographs [22, 49, 50] studying relationships between the environment and bicycle transport in the same age group. A detailed description of the manipulation process of the photographs and the choice tasks (good test-retest reliability > 70 %) [51] can be found elsewhere [30].

### Analyses

SPSS Statistics 22 was used to calculate the descriptive characteristics of the total sample. Conjoint analyses do not accommodate ‘typical’ moderation analysis, but they do allow latent class analysis to distinguish various subgroups according to their environmental preferences (i.e. importance of micro-environmental factors) for cycling for transport based on the choice-based conjoint tasks [47, 52]. Latent class analysis is a model-based approach where the cluster criterion choice is less arbitrary than the standard cluster analysis and shows a higher construct and predictive validity [53, 54]. Participants were assigned to a subgroup based on the highest probability of belonging to a class and not in a discrete manner (all-or-nothing) as with cluster analysis [55]. A latent class analysis with 15 replications was conducted in Sawtooth Software (SSI Web version 8.3.8.) [52]. The number of subgroups was selected based on the model fit, the number of participants in each subgroup and the distribution in the importance of the micro-environmental factors [52]. In Additional file 1, a detailed overview of the different models for 2, 3 and 4 subgroups is given. Finally, three subgroups emerged from our analysis of which the model had an Akaike’s Information Criterion (AIC) of 18755 and with a distribution of respectively 232, 598, and 1120 participants for each subgroup.

For each subgroup separately, Hierarchical Bayes (HB) estimation using dummy coding was executed to calculate part-worth utilities and importances [56]. The average relative importance represents the importance of each environmental factor on the preference for a street. These average importances are calculated by the difference in average part-worth utilities between the most and least preferred levels of a factor [47]. The average part-worth utilities symbolize the degree of preference given to a particular level of an environmental factor and can be interpreted similarly to a regression coefficient [47]. The greater the importance of an environmental factor, the greater the impact of that factor has on the choice. Furthermore, chi-square analyses (categorical variables) and MANOVAs (continuous variables) were performed in SPSS Statistics 22 to examine the significant differences in characteristics between the various subgroups. For all analyses, statistical significance was set at *p* < 0.05.

### Ethics, consent and permissions

The participants automatically gave their informed consent by filling in the online questionnaire. The study was approved by the Ethics Committee of Ghent University Hospital (B670201318588).

## Results

### Descriptive statistics of the total sample

The total sample consisted of 1950 participants aged between 45 and 65 years, 56.8 % were women, 64.6 % had undertaken tertiary education (college, university or postgraduate) and 21.7 % did not cycle for transport in a usual week. A detailed description of the total sample can be found in Table 1.

### Subgroup analysis – Differences in relative importance and part-worth utilities

Latent class analysis revealed three subgroups with homogenous preferences for the micro-environmental factors affecting the street’s appeal to cycle for transport. Table 2 presents the relative importance of each environmental factor within the total sample and the three subgroups. The corresponding part-worth utilities can be found in Additional file 2. Results indicated cycle path type was the most important micro-environmental factor for all participants. However, the importance of the other micro-environmental factors influencing the street’s appeal to cycle for transport varied across individuals, resulting in three subgroups.

**Table 2:** The relative importances of each environmental factor for the total sample and within each subgroup

	Total sample (*n* = 1950)^a^	Group 1 (*n* = 232)^a^	Group 2 (*n* = 598)^a^	Group 3 (*n* = 1120)^a^
Type of cycle path	58.47 ± 16.96	42.67 ± 5.12	39.44 ± 9.94	71.90 ± 4.10
Speed limit	8.29 ± 7.11	25.73 ± 3.95	4.10 ± 2.91	6.91 ± 2.45
Evenness of the cycle path	8.23 ± 5.96	7.50 ± 3.47	14.05 ± 7.22	5.27 ± 2.11
Traffic density	7.41 ± 7.45	7.47 ± 4.24	14.93 ± 9.01	3.38 ± 1.75
Vegetation	7.44 ± 5.31	7.33 ± 2.63	10.70 ± 7.72	5.72 ± 2.69
Maintenance	7.37 ± 6.20	5.12 ± 2.92	13.69 ± 7.30	4.46 ± 2.44
Speedbump	2.79 ± 2.23	4.17 ± 3.05	3.09 ± 2.41	2.35 ± 1.73

^a^Average relative importances % (M + SD)

Subgroup 1 consisted of 232 individuals. Following type of cycle path (42.7 %), this group attached most importance to stricter speed limits with an importance of 25.7 %. Next, the following three micro-environmental factors were less important than speed limit but did not significantly differ from each other: evenness of the cycle path (7.5 %), traffic density (7.5 %) and vegetation (7.3 %). Maintenance (5.1 %) and speedbumps (4.2 %) were the least important factors.

Subgroup 2 included 598 respondents and had a similar relative importance for type of cycle path (39.4 %) to subgroup 1. Following type of cycle path, traffic density (14.9 %), evenness of the cycle path (14.1 %) and maintenance (13.7 %) were the most important environmental factors. The importance of these environmental factors did not differ significantly. Vegetation (10.7 %) and speed limits (4.1 %) were significantly less important. Finally, speed bump had the lowest importance (3.1 %).

Subgroup 3 represented the largest group with 1120 participants and attached relatively more importance to type of cycle path (71.9 %) compared to both other subgroups. The other micro-environmental factors were significantly less important: speed limits (6.9 %), vegetation (5.7 %), evenness of the cycle path (5.3 %), maintenance (4.5 %), traffic density (3.4 %) and speed bump (2.4 %).

### Subgroup analysis – Differences in characteristics between the three subgroups

Descriptive characteristics and significant differences in socio-demographics, transport behavior, psychosocial determinants of cycling for transport, neighborhood environmental perceptions, cycling skills, concerns and preferences between the three subgroups can be found in Table 1. A significant difference was found for gender with subgroup 2 having a larger proportion of women. Area of residence also differed significantly between the three subgroups: 54.3 % of the participants of subgroup 1 lived in a village, town or rural area, compared to 56.7 % of subgroup 2 and 62.0 % of subgroup 3. No significant differences for age, SES, BMI and cohabitation were found between the three subgroups.

Results from the MANOVAs showed that minutes of cycling for transport per week, minutes of walking for transport per week and number of motorized vehicles were significantly different between subgroup 1 and the two other subgroups. Participants of subgroup 1 cycled and walked significantly more for transport and owned significantly less motorized vehicles compared to subgroup 2 or subgroup 3 (no significant difference between subgroup 2 and 3). No significant differences between the three subgroups were found for minutes of motorized transport and bicycle leisure time per week.

Differences in psychosocial determinants between the three subgroups were found for habit, modeling, self-efficacy, perceived benefits and perceived barriers. Subgroup 1 showed significantly higher scores on habit, self-efficacy, perceived benefits and a lower score on perceived barriers compared to subgroup 2 and subgroup 3. Additionally, subgroup 1 perceived significantly more modeling compared to subgroup 3. No significant differences were found for social norm and social support between the three subgroups.

There was only one significant difference in neighborhood environmental perceptions between the subgroups. Subgroup 1 perceived significantly less single unit houses in the neighborhood compared to subgroup 2 and subgroup 3.

Significant differences in cycling concerns and preferences were found for “as a cyclist I feel vulnerable in the traffic” and for “I prefer the safest cycling route”. Subgroup 2 reported lower preference for the safest route and felt less vulnerable in traffic compared to both other subgroups. Furthermore, subgroup 3 reported a higher preference for cycling alone in comparison to subgroup 1 and 2. Finally, subgroup 3 assigned more importance to wearing a fluorescent vest or bicycle helmet than subgroup 2. No significant differences were found between the three subgroups for cycling skills and the other cycling preferences (preferring the shortest or most beautiful cycling route).

## Discussion

To target at risk subgroups regarding cycling for transport, the different needs of particular subpopulations need to be identified. With latent class analysis, three subgroups of the middle-aged adult population could be distinguished. These subgroups had similar preferences for micro-environmental characteristics based on the responses given to a series of choice tasks depicting potential cycling routes. Previously, we showed that the provision of cycle paths separated from motorized traffic was the best strategy to increase a street’s appeal to cycle for transport in a population sample [30]. Results from the present study indicated that type of cycle path remained the most important environmental factor for all three subgroups, but significant differences in preferences for the other micro-environmental factors were observed.

The first subgroup distinguished itself from the other subgroups by awarding relatively more importance to restrictions in speed limits and being the most physically active group compared to both other subgroups. It had significantly higher rates of walking and cycling for transport, owned significantly less motorized vehicles and perceived less single unit houses in their neighborhood environment. Additionally, subgroup 1 was characterized by a higher proportion of men and those living in urban places. Furthermore, this group reported more favorable values on psychosocial determinants of cycling for transport compared to both other subgroups, and perceived more modeling from partner, child (ren), friends or colleagues compared to subgroup 3. A possible explanation for the great importance regular cyclists attended to stricter speed limits, might be that they are more often confronted with the negative consequences of fast moving traffic (e.g. dangerous situations, noise, odor pollution) compared to someone who does not cycle regularly. Consequently, the presence of aesthetic and comfort-related environmental factors may be much less important for this subgroup, since traffic-related environmental factors predominate. Additionally, previous studies have found cycling for transport can be increased by increasing traffic safety through reducing the speed of motorized traffic [57, 58] and by increasing the speed of cyclists compared to the speed of cars [48, 59].

The second subgroup attached relatively more importance to traffic density, evenness of the cycle path, maintenance and vegetation. This subgroup consisted of the highest percentage of women, felt significantly less vulnerable in traffic and did not prefer the safest cycling route compared to both other subgroups. Since this subgroup cycled less than subgroup 1, it is important to know which environmental changes might increase the street’s appeal to encourage cycling for transport. A study of Twaddle et al. (2010) observed that women were more likely to be occasional cyclists, while men were more likely to be regular cyclists, and suggested if women’s cycling needs were tackled the number of cyclists could be increased [36]. Along with the finding that cycle path type also was most important in this subgroup, it seems that they mainly attached importance to traffic density, evenness of the cycle path, vegetation and maintenance, rather than speed limit or the presence of a speed bump. Consequently, interventions focusing on these factors, might offer a solution to increase the number or female cyclists. A possible explanation for these findings is that women attach more importance to the enjoyable aspect of cycling for transport than men [32, 36, 60].

Subgroup 3, representing the majority of all respondents, paid relatively more importance to cycle path type compared to both other subgroups and attached less importance to all other micro-environmental factors. This subgroup distinguished itself from subgroup 2 in that it attached more importance to wearing a fluorescent vest or a helmet. Furthermore, this group showed the least preference to cycle alone and contained the highest percentage of inhabitants living in a rural environment, village or town of all groups. The higher importance score for cycle path type in subgroup 3 might be explained by their lower preference for cycling alone than both other subgroups, and therefore might give more attention to features enabling cycling with other people side by side. Furthermore, in less-urbanized environments speed limits are often less strict compared to urban environments [61], and consequently it might be that people living in a rural environment attach more importance to being well separated from the fast-moving traffic.

In conclusion, it can be assumed that subgroup 2 and subgroup 3 could be seen as at risk populations (e.g. together 88.1 % of the sample) since they cycled significantly less in comparison to subgroup 1. Cycle path type appeared to be by far the most important environmental factor in comparison to the other micro-environmental factors, and certainly for subgroup 3, representing the majority of the respondents. The most preferred cycle path type was a cycle path separated from motorized traffic by a hedge (hedge > curb > white lines) and might be further improved by a separation from the sidewalk by color [30]. However, the effect of separation from the sidewalk was much less pronounced than separation from motorized traffic. This is in line with the results from Winters et al. (2010) who found consistent results supporting the importance of separated cycle paths from traffic independent of the type of cyclist (regular, frequent, occasional and potential cyclists) [62]. In addition, a stated preference study indicated that cycling facilities separated from motorized traffic were the most preferred form of cycling infrastructure, regardless of cycling confidence [63]. Therefore, we can conclude that no tailoring is required for an intervention that focuses on better separated cycle paths, since it is by far the most important factor for all subgroups. Apart from this result, in situations when a good separated cycle path is already provided or cannot be provided, no clear difference was found between the relative importance of the other micro-environmental factors for subgroup 2 and 3. Broadly speaking, all other factors (i.e. traffic density, evenness of the cycle path, maintenance, vegetation and speed limits) seem similarly important (i.e. there was no consistent pattern of difference in importances) and that providing a speed bump will have the least impact on the street’s appeal to cycle for transport. Consequently, results from our study can advise developers of environmental interventions with the purpose of encouraging cycling for transport that tailored interventions in this context are not needed. Nevertheless, future research in real life settings is warranted to investigate if changes to micro-environmental factors are associated with changes in actual cycling behavior among particular subgroups.

A strength of the current study is the use of latent class analysis to investigate whether specific subgroups exist based on similarities in micro-environmental preferences to cycle for transport. Latent class analysis is a model-based clustering approach which means that the cluster criterion choice is less arbitrary than the standard cluster analysis and shows a higher construct and predictive validity [53, 54, 64]. Furthermore, a large sample of 1950 middle-aged adults could be reached by distributing the research through the web which allowed comparable numbers in each subgroup. Nevertheless, this web-based sampling method has also some limitations such as the overrepresentation of certain individuals (e.g. 64.6 % had a tertiary education and 78.3 % did cycle for transport in a usual week) in comparison to the statistics of the Flemish population where 28.1 % has a tertiary education [65] and around 45 % indicate using their bicycle weekly [66]. Therefore, caution is needed when generalizing the present results to the entire middle-aged Flemish population. Furthermore, the most important limitation is that the current study did not assess effects on actual cycling behavior, but only the street’s appeal to cycle for transport. Consequently, these findings need to be confirmed by on-site research.

## Conclusions

Although latent class analysis revealed three different subgroups in the middle-aged adult population based on their environmental preferences, results indicated that cycle path type (i.e. a good separated cycle path) is the most important environmental factor for all participants and certainly for individuals who did not cycle for transport. Furthermore, only negligible differences were found between the relative importance of the other micro-environmental factors (i.e. traffic density, evenness of the cycle path, maintenance, vegetation and speed limits) and that providing a speed bump has the least impact on a street’s appeal to cycle for transport. This suggests that tailored environmental interventions are not needed in this research context.

## Abbreviation

PA, physical activity

## Additional files

Additional file 1: A detailed overview of the different models for 2, 3 and 4 subgroups. (PDF 128 kb) Additional file 2: The part worth utilities of the environmental levels of each factor separately for each subgroup. (PDF 53 kb)
