# Supplementary material for: Differences in environmental preferences towards cycling for transport among adults: a latent class analysis
Source: BMC Public Health. 2016 Aug 12;16:782. doi: 10.1186/s12889-016-3471-5 (PMC4983031; doi:10.1186/s12889-016-3471-5)
Supplement: Additional file 1: — A detailed overview of the different models for 2, 3 and 4 subgroups. (PDF 128 kb) [file 12889_2016_3471_MOESM1_ESM.pdf]

Additional file 1

Table 1.1. Summary of best replications

Groups	Log-likelihood	Pct Cert	AIC	CAIC	BIC	ABIC	Chi-Square	Relative Chi-Square
2	-9459	41,68	18980	19260	19229	19131	13522	436
3	-9331	42,47	18755	19181	19134	18985	13778	293
4	-9245	43,00	18617	19187	19124	18924	13949	221

Table 1.2. Tabulation with the comparison between different groups

Tabulation of 2 group vs. 3 group solutions

	1	2	3	Total
1	179	593	38	810
2	53	5	1082	1140
Total	232	598	1120	1950

Tabulation of 3 group vs. 4 group solutions

	1	2	3	4	Total
1	40	2	2	188	232
2	362	0	222	14	598
3	239	868	0	13	1120
Total	641	870	224	215	1950

Table 1.3. The distribution in attribute importances for 2, 3 and 4 groups

**Attribute Importances (%) 2 groups**		
Type of cycle path	42,5	73,8
Speed limits	8,8	8,4
Speed bump	1,4	2,0
Vegetation	9,6	5,2
Evenness of cycle path	12,5	4,8
Maintenance	11,9	4,1
Traffic density	13,2	1,9

The average maximum membership probability is 0.89072.

**Attribute Importances (%) 3 groups**			
Type of cycle path	47,8	41,41	74,3
Speed limits	24,5	2,9	6,9
Speed bump	3,6	0,7	1,9
Vegetation	7,5	10,7	5,5
Evenness of cycle path	5,7	15,1	5,1
Maintenance	4,0	14,4	4,3
Traffic density	6,8	14,8	2,0

The average maximum membership probability is 0.84138.

**Attribute Importances (%) 4 groups**				
Type of cycle path	60,8	75,81102	24,45952	48,10116
Speed limits	3,8	8,26820	3,75293	25,73029
Speed bump	1,0	2,0	0,6	3,8
Vegetation	8,8	4,6	11,7	7,2
Evenness of cycle path	10,9	4,2	17,7	3,9
Maintenance	8,1	3,7	19,5	4,4
Traffic density	6,5	1,5	22,3	6,9

The average maximum membership probability is 0.77612.
