# Supplementary material for: Differences in environmental preferences towards cycling for transport among adults: a latent class analysis
Source: BMC Public Health. 2016 Aug 12;16:782. doi: 10.1186/s12889-016-3471-5 (PMC4983031; doi:10.1186/s12889-016-3471-5)
Supplement: Additional file 2: — The part worth utilities of the environmental levels of each factor separately for each subgroup. (PDF 53 kb) [file 12889_2016_3471_MOESM2_ESM.pdf]

Additional file 2

Table 2.1: The part worth utilities of the environmental levels of each factor separately for each subgroup.

	Subgroup 1			Subgroup 2			Subgroup 3		
	MEAN	95% CI-	95% CI+	MEAN	95% CI-	95% CI+	MEAN	95% CI-	95% CI+
**Type of cycle path**									
C1	0.00	0.00	0.00	0.00	0.00	0.00	0.00	0.00	0.00
C2	6.05	5.88	6.22	7.44	7.32	7.56	10.98	10.89	11.06
C3	9.10	8.93	9.26	8.34	8.25	8.43	16.09	16.04	16.15
C4	13.27	13.01	13.53	8.83	8.71	8.95	24.72	24.64	24.80
C5	10.51	10.37	10.65	8.68	8.58	8.79	18.50	18.46	18.55
C6	12.83	12.62	13.03	9.25	9.02	9.48	25.73	25.68	25.78
**Speed limits**									
50 km/h	0.00	0.00	0.00	0.00	0.00	0.00	0.00	0.00	0.00
30 km/h	8.18	8.05	8.32	0.64	0.56	0.73	2.50	2.44	2.55
**Speedbump**									
absent	0.00	0.00	0.00	0.00	0.00	0.00	0.00	0.00	0.00
present	1.17	1.01	1.32	0.15	0.08	0.22	0.60	0.55	0.65
**Vegetation**									
no trees	0.00	0.00	0.00	0.00	0.00	0.00	0.00	0.00	0.00
two trees	2.10	1.98	2.23	1.63	1.55	1.72	1.23	1.19	1.27
four trees	1.35	1.17	1.52	2.18	2.01	2.34	1.94	1.87	2.01
**Evenness of the cycle path**									
very uneven	0.00	0.00	0.00	0.00	0.00	0.00	0.00	0.00	0.00
moderately uneven	-0.74	-0.94	-0.54	0.96	0.83	1.09	0.62	0.57	0.66
even	0.90	0.62	1.18	3.37	3.21	3.52	1.76	1.71	1.82
**Maintenance**									
bad maintenance	0.00	0.00	0.00	0.00	0.00	0.00	0.00	0.00	0.00
moderate maintenance	0.57	0.39	0.74	2.24	2.16	2.33	0.90	0.87	0.94
good maintenance	0.73	0.57	0.88	3.20	3.03	3.38	1.41	1.35	1.48
**Traffic density**									
4 cars + truck	0.00	0.00	0.00	0.00	0.00	0.00	0.00	0.00	0.00
3 cars	1.15	0.93	1.37	2.23	2.12	2.33	0.12	0.08	0.16
1 car	1.69	1.44	1.95	3.45	3.25	3.65	0.78	0.72	0.84

C1: no cycle path; C2: cycle path separated from traffic by marked white lines; C3: cycle path separated from traffic with a curb, not separated from walking path by color; C4: cycle path separated from traffic with a hedge, not separated from walking path by color; C5: cycle path separated from traffic with a curb, separated from walking path by color; C6: cycle path separated from traffic with a hedge, separated from walking path by color.
